# Salivary Small Extracellular Vesicles Associated miRNAs in Periodontal Status—A Pilot Study

**DOI:** 10.3390/ijms21082809

**Published:** 2020-04-17

**Authors:** Pingping Han, Peter Mark Bartold, Carlos Salomon, Saso Ivanovski

**Affiliations:** 1 School of Dentistry, The University of Queensland, Brisbane, QLD 4006, Australia; p.han@uq.edu.au; 2 School of Dentistry, The University of Adelaide, Adelaide, SA 5000, Australia; mark.bartold@adelaide.edu.au; 3 Exosome Biology Laboratory, Centre for Clinical Diagnostics, the University of Queensland Centre for Clinical Research, Royal Brisbane and Women’s Hospital, The University of Queensland, Brisbane, QLD 4029, Australia; c.salomongallo@uq.edu.au; 4 Department of Obstetrics and Gynecology, Ochsner Baptist Hospital, New Orleans, LA 70422, USA; 5 Department of Clinical Biochemistry and Immunology, Faculty of Pharmacy, University of Concepción, Concepción 4030000, Chile

**Keywords:** salivary small extracellular vesicles, size exclusion chromatography, miRNAs, periodontal disease, biomarker discovery

## Abstract

This pilot study aims to investigate whether salivary small extracellular vesicle (sEV)-associated microRNAs could act as potential biomarkers for periodontal disease status. Twenty-nine participants (10 who were healthy, nine with gingivitis, 10 with stage III/IV periodontitis) were recruited and unstimulated whole saliva samples were collected. Salivary sEVs were isolated using the size-exclusion chromatography (SEC) method and characterised by morphology, EV-protein and size distribution using transmission electron microscopy (TEM), Western Blot and Nanoparticle Tracking Analysis (NTA), respectively. Ten mature microRNAs (miRNAs) in salivary sEVs and saliva were evaluated using RT-qPCR. The discriminatory power of miRNAs as biomarkers in gingivitis and periodontitis versus healthy controls was evaluated by Receiver Operating Characteristics (ROC) curves. Salivary sEVs were comparable to sEVs morphology, mode, size distribution and particle concentration in healthy, gingivitis and periodontitis patients. Compared to miRNAs in whole saliva, three significantly increased miRNAs (hsa-miR-140-5p, hsa-miR-146a-5p and hsa-miR-628-5p) were only detected in sEVs in periodontitis when compared to that of healthy controls, with a good discriminatory power (area under the curve (AUC) = 0.96) for periodontitis diagnosis. Our study demonstrated that salivary sEVs are a non-invasive source of miRNAs for periodontitis diagnosis. Three miRNAs that are selectively enriched in sEVs, but not whole saliva, could be potential biomarkers for periodontal disease status.

## 1. Introduction

Periodontitis is a complex inflammatory disease, associated with a dysbiotic plaque biofilm and characterised by the destruction of periodontal tissues. Currently, it is clinically diagnosed by clinical attachment loss (CAL), periodontal pocket depth (PPD), bleeding on probing (BOP) and radiographic bone loss. Aside from the fact that these parameters are mostly measures of past disease activity and, in the case of BOP, have poor predictive properties for disease progression, they also require professional dental assessment. Given the widespread incidence of periodontitis in approximately 50% of the global adult population [1], there is a plausible rationale for the development of a biologically based non-invasive diagnostic system for periodontal disease status.

Human saliva is an attractive source of biomarkers for periodontitis; it is easy to access through non-invasive means, is low-cost, and potentially provides a “mirror” of the periodontal status of a patient [2,3]. Salivary levels of pro-inflammatory cytokines [4,5], chemokines [6], matrix metalloproteinases [7,8] and bone remodelling proteins [4,9,10,11] have been investigated for their ability to distinguish between individuals with periodontal disease and those who are healthy. However, most proteins are present in low concentrations in saliva and have a limited diagnostic value due to their poor sensitivity and specificity [11].

In the past 10 years, the role of extracellular vesicles (EVs) in cell-to-cell communication has been widely explored [12]. EVs are lipid-encapsulated vesicles with the capacity to transport bioactive molecules (i.e., microRNAs (miRNAs)) which can be delivered to other cells to regulate their biological function. There are several types of EVs, which can be classified by their sizes, into small EVs (i.e., sEVs, exosomes), medium (i.e., microvesicles) and large EVs (i.e., macrovesicles and apoptotic bodies). Currently, there is an ongoing discussion in the literature about the heterogeneity of EVs and the correct nomenclature; however, the majority of studies have focused on small EVs such as exosomes. sEVs (< 200 nm), which are abundant in saliva and are emerging as a potential source for the development of diagnostic tools for a variety of diseases [13,14,15,16], owing to their components—nucleic acids (microRNAs, DNAs and other RNAs), lipids and proteins. Salivary sEVs have not been widely explored as a diagnostic tool in periodontology, probably because the methodology for their isolation is still developing. Very recent research has demonstrated that salivary CD9- and CD81-positive (two tetraspanin proteins enriched in EVs) sEVs are decreased in periodontitis compared to healthy controls [17]. Other recent research has demonstrated that the salivary sEV (exosomal) programmed death-ligand 1 (PD-L1) mRNA is significantly increased (*p* < 0.01) in periodontitis patients versus non-periodontitis subjects [18].

sEVs can carry a wide range of bioactive molecules and are enriched in small non-coding RNAs [19]. MicroRNAs (miRNAs) are non-coding RNAs, 19–25 nucleotides in length. Very recent research has demonstrated that miR-512-3p and miR-412-3p were upregulated in salivary sEVs from oral squamous cell carcinoma patients compared to the controls [20]. However, no studies have explored miRNAs’ expression patterns in salivary sEVs as potential biomarkers for periodontal status. According to published reviews [21,22,23,24,25,26], ten periodontitis-associated miRNAs (hsa-miR-15a-5p, hsa-miR-29b-3p, hsa-miR-124-3p, hsa-miR-140-5p, hsa-miR-146a-5p, hsa-miR-148a-3p, hsa-miR-155-5p, hsa-miR-223-3p, hsa-miR-301b, hsa-miR-628-5p) have been explored as potential periodontitis biomarkers from one or more sample sources, such as saliva, gingival tissues, or periodontium-derived cells. However, since miRNAs are expressed in a tissue and biofluid-specific manner, it is important to investigate whether these proposed miRNA periodontitis biomarker candidates are also expressed in salivary sEVs. Additionally, whether salivary sEV-associated miRNAs have the same profile as whole saliva-associated miRNAs in periodontitis remains unknown.

This pilot study aimed to evaluate the miRNA expression profile of whole saliva and salivary sEVs obtained from gingivitis, periodontitis and periodontally healthy patients, and to determine the diagnostic potential of miRNAs associated with sEVs as biomarkers of periodontal status.

## 2. Results

### 2.1. Demographic and Clinical Characteristics of the Study Groups

As shown in Table 1, the participants (*n* = 29) were from various ethnic backgrounds (caucasian, Asian, and others) and mixed-gender (20 males, nine females), with ages ranging from 23 to 75. It is noted that most of the participants are non-smokers and there is an age difference between non-periodontitis and periodontitis patients.

### 2.2. Salivary sEVs Characteristics

**Figure 1:**
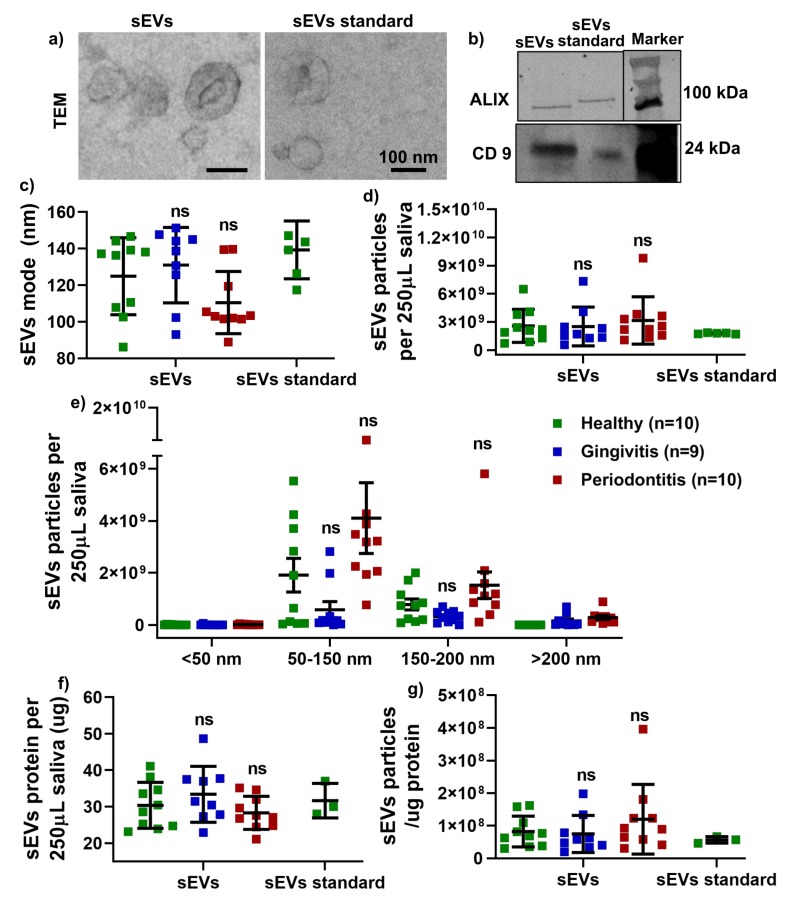
Characterisation of isolated salivary small extracellular vesicles (sEVs) by morphology using transmission electron microscopy (TEM) (**a**), by sEV-related proteins (ALIX and CD9) using Western blotting (WB) (**b**) and by particle mode (**c**), concentration (**d**), size distribution using Nanoparticle Tracking Analysis (NTA) (**e**), protein (**f**) and particle/ug protein (**g**). No significant difference vs healthy group (ns).

Transmission electron microscopy (TEM) analysis confirmed that the salivary sEVs were cup-shaped (Figure 1a) and Western Blot analysis demonstrated the expression of the sEV-associated proteins ALG-2 interacting protein X (ALIX) and CD9 (Figure 1b). Nanoparticle Tracking Analysis (NTA) results showed that the sEVs’ average modes (Figure 1c) and particle numbers (Figure 1d) were comparable between the healthy, gingivitis and periodontitis groups. Regarding the size distribution, it was noted that the sEV concentration for the size range of 50–150 and 150–200 nm was slightly increased in the periodontitis patients compared to that of the healthy controls; however, the difference was not statistically significant (Figure 1e). There was no significant difference in terms of total protein for salivary sEVs (f) and the ratio of salivary sEV particles/total protein (g) between the healthy, gingivitis and periodontitis groups.

### 2.3. miRNAs Expression Pattern in sEVs and Saliva

**Figure 2:**
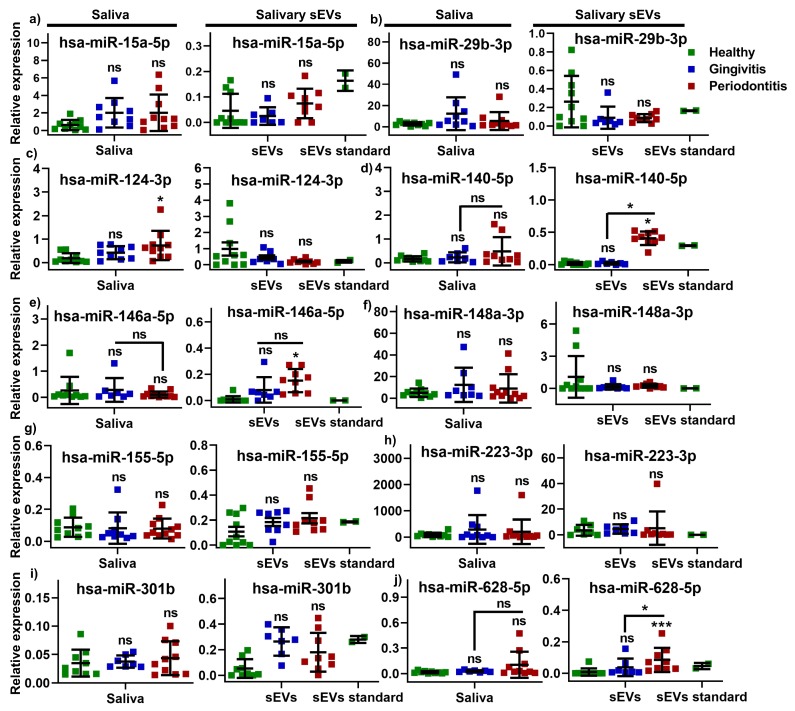
The relative expression levels of microRNAs (miRNAs): hsa-miR-15a-5p (**a**), hsa-miR-29b-3p (**b**), hsa-miR-124-3p (**c**), hsa-miR-140-5p (**d**), hsa-miR-146a-5p (**e**), hsa-miR-148a-3p (**f**), hsa-miR-155-5p (**g**), hsa-miR-223-3p (**h**), hsa-miR-301b (**i**) and hsa-miR-628-5p (**j**) miRNA detected by qRT-PCR in salivary sEVs and saliva samples from gingivitis, periodontitis patients, compared to healthy subjects. No significant difference vs healthy group (ns). * *p* < 0.05 vs healthy group; *** *p* < 0.0002 vs healthy control.

The expression of 10 miRNAs (hsa-miR-15a-5p, hsa-miR-29b-3p, hsa-miR-124-3p, hsa-miR-140-5p, hsa-miR-146a-5p, hsa-miR-148a-3p, hsa-miR-155-5p, hsa-miR-223-3p, hsa-miR-301b, hsa-miR-628-5p) was evaluated in salivary sEVs and whole unstimulated saliva in healthy, gingivitis and periodontitis patients (Figure 2). The analysis showed that three miRNAs (hsa-miR-140-5p (*p* < 0.05; Figure 2d), hsa-miR-146a-5p (*p* < 0.05; Figure 2e), hsa-miR-628-5p (*p* < 0.001; Figure 2j) were significantly upregulated in the salivary sEVs in periodontitis compared to the healthy controls. Both hsa-miR-140-5p (Figure 2d) and hsa-miR-628-5p (Figure 2j) showed a significant difference (*p* < 0.05) in salivary sEVs between gingivitis and periodontitis. No statistically significant differences were found between the healthy and gingivitis groups for any of the miRNAs that were assessed. In the saliva-only comparison, only hsa-miR-124-3p (Figure 2c) was significantly increased (*p* < 0.05) in the periodontitis compared to the healthy groups. All of the other miRNAs showed no statistically significant changes in either sEVs or saliva samples between the healthy, gingivitis and periodontitis groups.

### 2.4. Discriminatory Power of Upregulated miRNAs in Salivary sEVs

**Figure 3:**
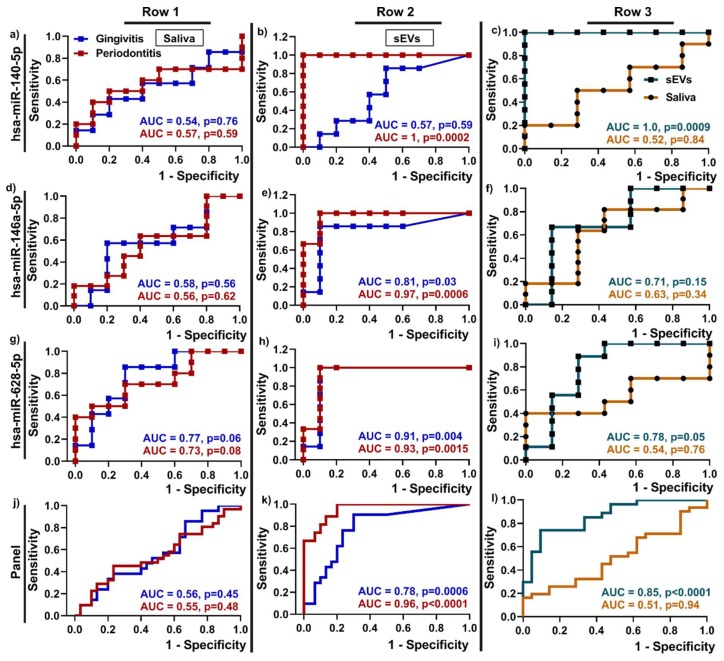
Discriminatory power of upregulated miRNAs hsa-miR-140-5p (**a**–**c**), hsa-miR-146a-5p (**d**–**f**), hsa-miR-628-5p (**g**–**i**) and the miRNA panel (**j**–**l**) in salivary sEVs from periodontally healthy, gingivitis and periodontitis patients by using Receiver Operating Characteristics (ROC) curves and area under the curve -AUC. Note: Row 1 and 2 show discriminatory power between periodontitis or gingivitis vs periodontal health of whole saliva miRNA (**a**, **d**, **g**, **j**) and sEV mRNA (**b**, **e**, **h**, **k**); Row 3 (**c**, **f**, **i**, **j**) shows the discriminatory power of upregulated miRNAs in saliva and sEVs between gingivitis and periodontitis patients.

Receiver Operating Characteristics (ROC) curves were used to examine the discriminatory power of the three upregulated miRNAs as potential biomarkers for periodontitis compared to healthy patients (Figure 3). The ROC curves are representative of the sensitivity (true positive rate: Y-axis) and one-specificity (false positive rate: X-axis), while the area under the curve (AUC) indicates the discriminatory power of the biomarkers.

The data show that hsa-miR-140-5p (AUC = 1, b), hsa-miR-146a-5p (AUC = 0.97, e), hsa-miR-628-5p (AUC = 0.93, h) in salivary sEVs performed better in discriminating between periodontitis and health compared to saliva (AUC = 0.57, 0.56, 0.73 respectively) (Figure 3a,d,g). Moreover, hsa-miR-146a-5p (AUC = 0.81, e) and hsa-miR-628-5p (AUC = 0.91, h) in salivary sEVs showed the same trend in gingivitis compared to healthy patients. The panel of all three miRNAs showed a high AUC value at 0.96 in sEVs for periodontitis and a moderate AUC value at 0.78 in sEVs for gingivitis, compared to the healthy controls (Figure 3d).

Comparing gingivitis and periodontitis (c, f, i, l), all three miRNAs in sEVs performed better in discriminating between gingivitis and periodontitis, compared to saliva.

## 3. Discussion

This pilot study is the first to investigate the diagnostic potential of salivary sEV-associated miRNAs in periodontal disease. The overall objective was to determine the diagnostic value of miRNAs obtained from sEVs compared to those sourced from whole saliva in terms of discriminating between different periodontal status. It was shown that three miRNAs (hsa-miR-140-5p, hsa-miR-146a-5p, hsa-miR-628-5p) were highly significantly upregulated (*p* < 0.05) in salivary sEVs from periodontitis compared to healthy controls, with all three showing high discriminatory power at AUC > 0.9, which suggests that they are potential biomarkers for periodontitis. Conversely, in the whole saliva samples, only one miRNA showed a moderately statistically significant increase (*p* < 0.05) in periodontitis compared to healthy patients, and the discriminatory power of this sole miRNA was low. Therefore, it appears that miRNAs sourced from sEVs have superior potential compared to whole saliva samples as diagnostic biomarkers of periodontal disease status.

The utilization of sEVs for both therapeutic and diagnostic applications is a rapidly developing field, with continual refinement of isolation methods. In particular, stem cell-derived sEVs have been explored as a promising cell-free regenerative medicine strategy for bone tissue engineering [27,28]. The two published studies reporting on salivary sEV research in the periodontal field used a precipitation-based ExoQuick kit [17,18], which has the potential to produce protein and nucleic acid contamination [29]. Size exclusion chromatography (SEC) has been proposed for sEV isolation with high purity and higher functionality [30], and was applied in the current study. It should also be noted that the current study also addressed several shortcomings noted in the existing literature investigating sEVs for periodontal diagnostic purposes [17,18], as the sEVs in the current study were appropriately characterised according to the newly published guidelines [31].

MicroRNAs (miRNAs) are a group of endogenous non-coding small RNAs, 19–25 nucleotides in length, that play an important role in development, cell differentiation, proliferation and survival [32]. A single miRNA is capable of suppressing the translation of hundreds of genes by targeting the specific 3′-UTR regions of their mRNAs. Thus, there is now compelling evidence that miRNAs are associated with physiological and pathological process that contribute to the development and progression of systemic diseases, such as cancers [33] and vascular disease [34], as well as periodontal disease [22,35]. Several studies described the potential of miRNAs as putative diagnostic biomarkers for periodontal disease, including hsa-miR-15a-5p, hsa-miR-29b-3p, hsa-miR-124-3p, hsa-miR-140-5p, hsa-miR-146a-5p, hsa-miR-148a-3p, hsa-miR-155-5p, hsa-miR-223-3p, hsa-miR-301b, hsa-miR-628-5p [21,22,23,24,25,26]. It has been reported that hsa-miR-140-5p and hsa-miR-628-5p in gingival crevicular fluid are potential diagnostic biomarkers for periodontitis [25]. Evidence demonstrated hsa-miR-146a-5p might serve as an indicator for periodontal disease severity in both gingival tissues [36] and saliva [37]. However, no report has described miRNA expression in salivary sEVs from healthy, gingivitis and periodontitis patients. Our RT-qPCR results demonstrated that hsa-miR-140-5p, hsa-miR-146a-5p and hsa-miR-628-5p were only significantly enriched in salivary sEVs, but not whole saliva, from periodontitis samples compared to controls. Interestingly, both hsa-miR-140-5p and hsa-miR-628-5p in salivary sEVs, but not whole saliva, were able to differentiate between gingivitis and periodontitis (*p* < 0.05 in Figure 2d and Figure 3e). These data on salivary sEVs are in line with other recent reports [26,36,37].

To better evaluate the discriminatory power of the upregulated miRNAs as gingivitis and periodontitis biomarkers (compared to periodontal health), we generated ROC curves. Notwithstanding the limited patient numbers, our data showed the high discriminatory power of hsa-miR-140-5p, hsa-miR-628-5p and hsa-miR-146a-5p, where all three miRNAs resulted in a high AUC value (AUC > 0.9) for periodontitis diagnosis, indicating that these three miRNAs in salivary sEVs are good predictors and can be suggested as new candidate biomarkers for periodontitis. Notably, the results from this study indicate that hsa-miR-140-5p and hsa-miR-146a-5p in sEVs can discriminate between healthy and periodontitis patients only, while hsa-miR-628-5p could be potential biomarkers for both gingivitis and periodontitis, compared to healthy controls. These three miRNAs as a panel may be a good indicator for periodontal disease (gingivitis and/or periodontitis). Moreover, hsa-miR-140-5p (AUC = 1 in Figure 3c) in sEVs (but not whole saliva) could be a potential marker to discriminate between gingivitis and periodontitis patients.

These findings suggest that sEVs may provide a superior diagnostic oral liquid biopsy, because they may be more enriched and more representative of the local environment compared to whole saliva, which is more influenced by systemic conditions. Notably, the periodontal status did not alter the sEVs’ modes, concentrations, protein quantities or size distribution.

As this was a pilot study with limited participant numbers, further studies will be required using larger sample sizes. The scarcity of research in this field precluded a meaningful sample size calculation, and the reported differences in the candidate biomarker miRNA expression in this pilot study can be used to calculate an appropriate sample size for future studies. A limitation of this study that needs to be addressed is the age discrepancy between the different groups, so that age can be removed as a potential confounding factor. Ideally, biomarkers that can discriminate with high sensitivity and specificity between health, gingivitis and periodontitis need to be identified. Small RNA deep sequencing would be helpful to determine more robust miRNA candidate biomarkers in salivary sEVs that can discriminate between periodontal statuses.

Taken together, based on the results and limitations in this study, our study used the SEC method to obtain enriched salivary sEVs and three upregulated miRNAs were detected in salivary sEVs from periodontitis patients only, compared to healthy patients.

## 4. Materials and Methods

### 4.1. Participants Recruitment and Sample Collection

Ethics approval for this study was obtained from The University of Queensland Human Ethics Committee (approval number: 2018001225, 12/11/2018). Healthy (*n* = 10), gingivitis (*n* = 9) and periodontitis (*n* = 10) participants were recruited with signed consent forms. As this was a pilot study, a sample size calculation was not undertaken, largely because there is a scarcity of data in this field for a meaningful calculation. Comprehensive periodontal examinations were performed by two independent periodontists for each participant to determine their periodontal pocket depths (PPD) and bleeding on probing (BOP). Periodontal status was defined based on recent guidelines [38] and according to the following criteria: a) healthy: without periodontal disease history; PPD < 4 mm; BOP < 20 % sites; b) gingivitis: generalised gingival inflammation without periodontal pocket, PPD < 4 mm; BOP > 20 % sites; c) stage III/IV periodontitis: > 30% of the sites with PPD with BOP and six sites PPD ≥ 6 mm on at least three teeth. All participants were non-smokers, had no underlying systemic diseases, and were not currently receiving any oral or periodontal treatment. The demographic and clinical characteristics of the study cohort are shown in Table 1.

Whole unstimulated saliva was collected from participants as described previously [39]. The participants were asked to refrain from eating and drinking for at least 1 hour prior to spitting the saliva samples into a tube and the samples were collected in the morning (9–12 a.m.). Fresh saliva samples were aliquoted and frozen in a −80 °C freezer.

### 4.2. Salivary sEVs Isolation

Commercially available SEC columns (miniPURE-EVs, HansaBioMed, Lonza, QLD, Australia) were used to fractionate the saliva sample according to the manufacturer’s protocol. Briefly, 250 μL of saliva was diluted in 250 μL of Dulbecco’s Phosphate Buffered Saline (DPBS, 1×, without Calcium, Magnesium, Phenol Red; In Vitro Technologies Pty Ltd, Australia), and centrifuged at 300 *g* for 15 minutes, 1600 *g* for 15 minutes, 16,000 *g* for 20 minutes at 4 °C. The supernatant was loaded on an SEC column and 100 μL fractions were collected. Fractions 7 to 11 were collected and concentrated to 100 μL using an Amicon Ultra 0.5 Centrifugal Filter Unit (10 kDa, Merck Millipore, QLD, Australia) by centrifugation at 14,000 *g* for 5 minutes at 4 °C.

A commercial lyophilised sEV (exosome) standard isolated from ‘healthy’ participants (Cat#: HBM-PESL; HansaBioMed, Lonza, QLD, Australia) was used as a control. However, the details of the ‘healthy’ participants’ recruitment process are unknown, and their periodontal health status may not have been determined. This sEV standard was isolated using the ultracentrifuge and ultrafiltration combination method.

### 4.3. Salivary sEVs Characterisation

Following the recommendations of the International Society of Extracellular Vesicles [31], sEVs were characterized by morphology, EV-associated protein markers, and size distribution, by TEM, Western Blot and NTA, respectively.

For the transmission electron microscopy (TEM) analysis, sEV samples were fixed in 3% (*w/v*) glutaraldehyde and analysed as previously described [40,41]. Briefly, 5 μL of each sample was adsorbed on Formvar carbon-coated and glow-discharged electron microscopy grids. After washing with PBS, the grids were transferred to a 50 μL drop of uranyl-oxalate solution, pH 7 for 3 mins. The grids were imaged using a FEI Tecan 12-transmission electron microscope (FEI, Hillsboro, OR).

Protein concentration in sEVs was determined using a Pierce BCA Protein Assay Kit (ThermoFisher Scientific, QLD, Australia) according to the manufacturer’s instructions. The samples (5 μL) were lysed with RIPA buffer and mixed with BCA working solution, followed by incubation at room temperature for 30 min. Absorbance was read at 562 nm on a Tecan Infinite M200 Pro spectrophotometer (Tecan, Switzerland, QLD, Australia).

Western blot, as previously described [42,43,44,45], was used to determine the sEV-associated proteins (CD 9 and ALIX). The sEV protein samples were separated by SDS-PAGE and transferred to polyvinylidene difluoride membrane using a Trans-Blot® Turbo™ Transfer System (BioRad, QLD, Australia). The membrane was blocked with Odyssey® Blocking Buffer at room temperature for 1 hour and primary antibodies (CD9, 1: 1000, Santa Cruz Biotechnology; ALIX, 1: 1000, Santa Cruz Biotechnology, QLD, Australia) were incubated overnight at 4 °C. After washing with 0.1% *v/v* Tween 20 in Tris-buffered saline (TBS), anti-rabbit DyLight 800 secondary antibody (1:10,000 in Odyssey Blocking Buffer, QLD, Australia) and anti-mouse DyLight 700 secondary antibodies were added and incubated for 1 h. The blots were visualised using a LI-COR Odyssey imaging system.

Nanoparticle tracking analysis was performed using a NanoSight NS500 instrument (NanoSight, United Kingdom) using a 488 nm laser module and NTA software version 3.1. Polystyrene latex beads (100 nm, Malvern NTA 4088) were used as a positive control and PBS was used as a negative control. Samples (5 μL) were diluted 1:160 with DPBS for the NS500 instrument to measure the rate of Brownian motion of nanoparticles in a light-scattering system. Five videos, each 30 s long were captured for each sample with a camera level of 14 and a detection threshold set to five. Each video file was processed and analysed to give the mean and mode of the particle sizes, along with the concentration and the number of particles.

### 4.4. RNA Extraction and miRNA Expression by RT-qPCR

Total RNA was isolated from ~10^9^ sEV particles using the Trizol method, following the manufacturer’s instructions and as described previously [39]. The quality and quantity of RNA were measured using a Tecan Infinite M200 Pro Spectrophotometer (TECAN, Australia).

Reverse transcription was performed using a miScript Reverse Transcription Kit (QIAGEN, Australia) and real-time PCR was performed using a miScript SYBRGreenKit (QIAGEN, Australia) and ABI StepOnePlus equipment. The primers for miRNAs (hsa-miR-15a-5p, hsa-miR-29b-3p, hsa-miR-124-3p, hsa-miR-140-5p, hsa-miR-146a-5p, hsa-miR-148a-3p, hsa-miR-155-5p, hsa-miR-223-3p, hsa-miR-301b, hsa-miR-628-5p) are listed in Table 2. The expression of each miRNA was normalized to the recommended housekeeping genes, human small nucleolar RNA SNORD48 [46] and hsa-miR-16-5p [47]. CT values >35 were excluded. ΔCT was calculated as the CTmiRNA target–CThousekeeping and the relative gene expression data were presented as 2^−ΔCT^.

### 4.5. Discriminatory Power Analysis

Receiver Operating Characteristic (ROC) curves and the area under the curve (AUC) were used to measure the discriminatory power of the upregulated miRNAs as biomarkers for periodontitis. For each upregulated miRNA, the ROC curves were generated using the relative gene expression levels of healthy controls and patients (gingivitis and periodontitis, respectively) by GraphPad Prism 8.3.1 software (San Diego, USA). Sensitivity, specificity, area under the curve (AUC, indicating the discriminatory power of the biomarkers) and p values, were calculated by the software.

### 4.6. Statistical Analysis

The data are presented as the mean ± SD (standard deviation). The data between healthy, gingivitis and periodontitis patients were determined by an ordinary one-way ANOVA analysis in Prism 8.3.1. ROC curves and AUC were determined by the Wilson/Brown method between healthy controls, and gingivitis and periodontitis, respectively. *p* < 0.05 was considered a statistically significant difference.

## Figures and Tables

**Table 1:** Demographic and clinical characteristics of the study cohort.

		Healthy (*n* = 10)	Gingivitis (*n* = 9)	Periodontitis (*n* = 10)
Gender	Male	6 (60%)	7 (78%)	7 (70%)
	Female	4 (40%)	2 (22%)	3 (30%)
Age		35.4 ± 2.4 (26–48)	31.8 5 ± 1.6 (23–40) *p* > 0.99	52.5 ± 4 (38–75) *p* = 0.01
Numbers of Teeth		27.1 ± 1.2	27.4 ± 1.1 *p* = 0.95	25.5 ± 3.4 *p* = 0.25
Smoking habit	Non-smokers	10 (100%)	10 (100%)	9 (90%)
	Smokers	0	0	1 (10%)
Ethnicity	Caucasians	5 (50%)	3	4 (40%)
	Asians	4 (40%)	5 (55%)	6 (60%)
	Others	1 (10%)	1	0
BOP		12.5% ± 1.14 (6–17%)	43.6% ± 5.33 (26–70%) *p* = 0.0001	43.7% ± 5.2 (20–71%) *p* < 0.0001
No. of deep pockets				31.3 ± 7.34 (5–66)
Average PPD (mm)			·	4.9 ± 0.4 (2.5–6.4)
Periodontitis classification	Localised (< 30%)			3 (30%)
	Generalised (≥30%)			7 (70%)
	Grade B			6 (60%)
	Grade C			4 (40%)
	Stage III			9 (90%)
	Stage IV			1 (10%)

Note: *p* values were calculated versus healthy controls.

**Table 2:** The miRNA primers used in this study.

	Forward Primer (5′ to 3′)
hsa-miR-15a-5p	TAGCAGCACATAATGGTTTGTGA
hsa-miR-29b-3p	CGCTAGCACCATTTGAAATCAG
hsa-miR-124-3p	ACGCGGTGAATGCCAAAA
hsa-miR-140-5p	CGCAGTGGTTTTACCCTATG
hsa-miR-146a-5p	TGAGAACTGAATTCCATGGGTTA
hsa-miR-148a-3p	CGCTCAGTGCACTACAGAACTTT
hsa-miR-155-5p	TGCTAATCGTGATAGGGGTAAA
hsa-miR-223-3p	TGTCAGTTTGTCAAATACCCCAAA
hsa-miR-301b	TGCAATGATATTGTCAAAGCAAA
hsa-miR-628-5p	CGATGCTGACATATTTACTAGAGGA
SNORD 48	CTCTGAGTGTGTCGCTGATGC
hsa-miR-16-5p	CGCCATAGCAGCACGTAAAT

## References

[1] Nazir M.A. (2017). Prevalence of periodontal disease, its association with systemic diseases and prevention. Int. J. Health Sci. (Qassim).

[2] Wren M.E., Shirtcliff E.A., Drury S.S. (2015). Not all biofluids are created equal: Chewing over salivary diagnostics and the epigenome. Clin. Ther..

[3] Pfaffe T., Cooper-White J., Beyerlein P., Kostner K., Punyadeera C. (2012). Diagnostic potential of saliva: Current state and future applications. Biochim. Clin..

[4] Buduneli N., Kinane D.F. (2011). Host-derived diagnostic markers related to soft tissue destruction and bone degradation in periodontitis. J. Clin. Periodontol..

[5] Kinane D.F., P P.M., Loos B.G. (2011). Host-response: Understanding the cellular and molecular mechanisms of hostmicrobial interactions: Consensus of the 7th European Workshop on Periodontology. J. Clin. Periodontol..

[6] Silva T.A., Garlet G.P., Fukada S.Y., Silva J.S., Cunha F.Q. (2007). Chemokines in oral inflammatory diseases: Apical periodontitis and periodontal disease. J. Dent. Res..

[7] Ebersole J.L., Schuster J.L., Stevens J., Dawson D., Kryscio R.J., Lin Y., Thomas M.V., Miller C.S. (2013). Patterns of salivary analytes provide diagnostic capacity for distinguishing chronic adult periodontitis from health. J. Clin. Immunol..

[8] Giannobile W.V., Al-Shammari K.F., Sarment D.P. (2003). Matrix molecules and growth factors as indicators of periodontal disease activity. Periodontology 2000.

[9] Kim J.J., Kim C.J., Camargo P.M. (2013). Salivary biomarkers in the diagnosis of periodontal diseases. J. Calif. Dent. Assoc..

[10] Zia A., Khan S., Bey A., Gupta N.D., Mukhtar-Un-Nisar S. (2011). Oral biomarkers in the diagnosis and progression of periodontal diseases. Biol. Med..

[11] Taylor J.J. (2014). Protein biomarkers of periodontitis in saliva. ISRN Inflamm..

[12] Paolicelli R.C., Bergamini G., Rajendran L. (2019). Cell-to-cell Communication by Extracellular Vesicles: Focus on Microglia. Neuroscience.

[13] Alharbi M., Zuñiga F., Elfeky O., Guanzon D., Lai A., Rice G.E., Perrin L., Hooper J., Salomon C. (2018). The potential role of miRNAs and exosomes in chemotherapy in ovarian cancer. Endocr.-Relat. Cancer.

[14] Salomon C., Nuzhat Z., Dixon C.L., Menon R. (2018). Placental Exosomes During Gestation: Liquid Biopsies Carrying Signals for the Regulation of Human Parturition. Curr. Pharm. Des..

[15] Sharma S., Zuñiga F., Rice G.E., Perrin L.C., Hooper J.D., Salomon C. (2017). Tumor-derived exosomes in ovarian cancer - liquid biopsies for early detection and real-time monitoring of cancer progression. Oncotarget.

[16] Edgar J.R. (2016). Q&A: What are exosomes, exactly?. BMC Biol..

[17] Tobon-Arroyave S.I., Celis-Mejía N., Córdoba-Hidalgo M.P., Isaza-Guzmán D.M. (2019). Decreased salivary concentration of CD9 and CD81 exosome-related tetraspanins may be associated with the periodontal clinical status. J. Clin. Periodontol..

[18] Yu J., Lin Y., Xiong X., Li K., Yao Z., Dong H., Jiang Z., Yu D., Yeung S.J., Zhang H. (2019). Detection of Exosomal PD-L1 RNA in Saliva of Patients With Periodontitis. Front. Genet..

[19] Cheng L., Sharples R.A., Scicluna B.J., Hill A.F. (2014). Exosomes provide a protective and enriched source of miRNA for biomarker profiling compared to intracellular and cell-free blood. J. Extracell. Vesicles.

[20] Gai C., Camussi F., Broccoletti R., Gambino A., Cabras M., Molinaro L., Carossa S., Camussi G., Arduino P.G. (2018). Salivary extracellular vesicle-associated miRNAs as potential biomarkers in oral squamous cell carcinoma. BMC Cancer.

[21] Irwandi R.A., Vacharaksa A. (2016). The role of microRNA in periodontal tissue: A review of the literature. Arch. Oral Biol..

[22] Luan X., Zhou X., Naqvi A., Francis M., Foyle D., Nares S. (2018). Diekwisch TGH MicroRNAs and immunity in periodontal health and disease. Int. J. Oral Sci..

[23] Olsen I., Singhrao S.K., Osmundsen H. (2017). Periodontitis, pathogenesis and progression: miRNA-mediated cellular responses to Porphyromonas gingivalis. J. Oral Microbiol..

[24] Schmalz G., Li S., Burkhardt R., Rinke S., Krause F., Haak R., Ziebolz D. (2016). MicroRNAs as Salivary Markers for Periodontal Diseases: A New Diagnostic Approach?. Biomed Res. Int..

[25] Kaczor-Urbanowicz K.E., Trivedi H.M., Lima P.O., Camargo P.M., Giannobile W.V., Grogan T.R., Gleber-Netto F.O., Whiteman Y., Li F., Li H.J. (2018). Salivary exRNA biomarkers to detect gingivitis and monitor disease regression. J. Clin. Periodontol..

[26] Saito A., Horie M., Ejiri K., Aoki A., Katagiri S., Maekawa S., Suzuki S., Kong S., Yamauchi T., Yamaguchi Y. (2017). MicroRNA profiling in gingival crevicular fluid of periodontitis-a pilot study. FEBS Open Bio.

[27] Trubiani O., Marconi G.D., Pierdomenico S.D., Piattelli A., Diomede F., Pizzicannella J. (2019). Human Oral Stem Cells, Biomaterials and Extracellular Vesicles: A Promising Tool in Bone Tissue Repair. Int. J. Mol. Sci..

[28] (2019). Periodontal Ligament Stem Cells: Current Knowledge and Future Perspectives. Stem Cells Dev..

[29] Zlotogorski-Hurvitz A., Dayan D., Chaushu G., Korvala J., Salo T., Sormunen R., Vered M. (2015). Human saliva-derived exosomes: Comparing methods of isolation. J. Histochem. Cytochem..

[30] Boing A.N., van der Pol E., Grootemaat A.E., Coumans F.A., Sturk A., Nieuwland R. (2014). Single-step isolation of extracellular vesicles by size-exclusion chromatography. J. Extracell. Vesicles.

[31] Théry C., Witwer K.W., Aikawa E., Alcaraz M.J., Anderson J.D., Andriantsitohaina R., Antoniou A., Arab T., Archer F., Atkin-Smith G.K. (2018). Minimal information for studies of extracellular vesicles 2018 (MISEV2018): A position statement of the International Society for Extracellular Vesicles and update of the MISEV2014 guidelines. J. Extracell. Vesicles.

[32] Bartel D.P. (2004). MicroRNAs: Genomics, biogenesis, mechanism, and function. Cell.

[33] Rapado-González Ó., Majem B., Muinelo-Romay L., Álvarez-Castro A., Santamaría A., Gil-Moreno A., López-López R., Suárez-Cunqueiro M.M. (2018). Human salivary microRNAs in Cancer. J. Cancer.

[34] Neumann A., Napp L.C., Kleeberger J.A., Benecke N., Pfanne A., Haverich A., Thum T., Bara C. (2017). MicroRNA 628-5p as a Novel Biomarker for Cardiac Allograft Vasculopathy. Transplantation.

[35] Fujimori K., Yoneda T., Tomofuji T., Ekuni D., Azuma T., Maruyama T., Mizuno H., Sugiura Y., Morita M. (2019). Detection of Salivary miRNAs Reflecting Chronic Periodontitis: A Pilot Study. Molecules.

[36] Ghotloo S., Motedayyen H., Amani D., Saffari M., Sattari M. (2019). Assessment of microRNA-146a in generalized aggressive periodontitis and its association with disease severity. J. Periodontal. Res..

[37] Gao Y., Hao C.D. (2018). Expression of miR-146a in saliva of chronic periodontitis patients and its influence on gingival crevicular inflammation and MMP-8/TIMP-1 levels. Shanghai Kou Qiang Yi Xue.

[38] Chapple I.L.C., Mealey B.L., Van Dyke T.E., Bartold P.M., Dommisch H., Eickholz P., Geisinger M.L., Genco R.J., Glogauer M., Goldstein M. (2018). Periodontal health and gingival diseases and conditions on an intact and a reduced periodontium: Consensus report of workgroup 1 of the 2017 World Workshop on the Classification of Periodontal and Peri-Implant Diseases and Conditions. J. Clin. Periodontol..

[39] Han P., Ivanovski S. (2019). Effect of Saliva Collection Methods on the Detection of Periodontium-Related Genetic and Epigenetic Biomarkers-A Pilot Study. Int. J. Mol. Sci..

[40] Dixon C.L., Richardson L., Sheller-Miller S., Saade G., Menon R. (2018). A distinct mechanism of senescence activation in amnion epithelial cells by infection, inflammation, and oxidative stress. Am. J. Reprod. Immunol..

[41] Thery C., Amigorena S., Raposo G., Clayton A. (2006). Isolation and characterization of exosomes from cell culture supernatants and biological fluids. Curr. Protoc. Cell Biol..

[42] Han P., Wu C.T., Xiao Y. (2013). The effect of silicate ions on proliferation, osteogenic differentiation and cell signalling pathways (WNT and SHH) of bone marrow stromal cells. Biomater. Sci..

[43] Han P., vanovski S., Crawford R., Xiao Y. (2015). Activation of the Canonical Wnt Signaling Pathway Induces Cementum Regeneration. J. Bone Miner Res..

[44] Han P., Wu C., Chang J., Xiao Y. (2012). The cementogenic differentiation of periodontal ligament cells via the activation of Wnt/beta-catenin signalling pathway by Li+ ions released from bioactive scaffolds. Biomaterials.

[45] Han P., Frith J.E., Gomez G.A., Yap A.S., O’Neill G.M., Cooper-White J.J. (2019). Five Piconewtons: The Difference between Osteogenic and Adipogenic Fate Choice in Human Mesenchymal Stem Cells. ACS Nano.

[46] Schwarzenbach H., da Silva A.M., Calin G., Pantel K. (2015). Data Normalization Strategies for MicroRNA Quantification. Clin. Chem..

[47] Masè M., Grasso M., Avogaro L., D’Amato E., Tessarolo F., Graffigna A., Denti M.A., Ravelli F. (2017). Selection of reference genes is critical for miRNA expression analysis in human cardiac tissue. A focus on atrial fibrillation. Sci. Rep..

